# Influence of Na Additives on the Characteristics of Titania-Based Humidity Sensing Elements, Prepared via a Sol–Gel Method

**DOI:** 10.3390/s25196075

**Published:** 2025-10-02

**Authors:** Zvezditza Nenova, Stephan Kozhukharov, Nedyu Nedev, Toshko Nenov

**Affiliations:** 1 Faculty of Electrical Engineering and Electronics, Technical University of Gabrovo, 4 H. Dimitar Str., 5300 Gabrovo, Bulgaria; nenova@tugab.bg (Z.N.); nnedev@tugab.bg (N.N.); 2 Center of Competence “Smart Mechatronic, Eco- and Energy-Saving Systems and Technologies”, 4 H. Dimitar Str., 5300 Gabrovo, Bulgaria; s.kozhukharov@uctm.edu; 3 Faculty of Chemical Technology, University of Chemical Technology and Metallurgy, 8 Kliment Ohridski Blvd., 1756 Sofia, Bulgaria

**Keywords:** humidity sensing elements, sol-gel method, titanium dioxide, sodium hydroxide, sodium tert-butoxide, Na additives

## Abstract

Humidity sensing elements based on sodium-doped titanium dioxide (Na-doped TiO_2_) were prepared using a sol–gel method in the presence of cerium ions and sintered at 400 °C and 800 °C. Titanium (IV) n-butoxide and a saturated solution of diammonium hexanitratocerate in isobutanol served as starting materials. Sodium hydroxide and sodium tert-butoxide were used as inorganic and organometallic sodium sources, respectively. The influence of sodium additives on the properties of the humidity sensing elements was systematically investigated. The surface morphologies of the obtained layers were examined by scanning electron microscopy (SEM). Elemental mapping was conducted by energy-dispersive X-ray (EDX) spectroscopy, and structural characterization was performed using X-ray diffractometry (XRD). Electrical properties were studied for samples sintered at different temperatures over a relative humidity range of 15% to 95% at 20 Hz and 25 °C. Experimental results indicate that sodium doping enhances humidity sensitivity compared to undoped reference samples. Incorporation of sodium additives increases the resistance variation range of the sensing elements, reaching over five orders of magnitude for samples sintered at 400 °C and four orders of magnitude for those sintered at 800 °C.

## 1. Introduction

Humidity is important for humans and the activities they perform. Too high or too low humidity has an adverse effect on humans. Humidity is also a factor in various technological processes. Therefore, humidity measurement and control are of great importance and are increasingly being used in industry and agriculture, meteorology, medicine and healthcare, environmental monitoring, and to ensure healthy and safe working conditions, among other applications [1,2,3].

Humidity characterizes the amount of water vapor in the atmosphere. As a standard for determining humidity values, relative humidity (*RH*) is the most widely applied [4]. Depending on the conversion principle, various types of humidity sensors are used. These include resistive and capacitive sensors [4,5,6], as well as sensors based on optical fibers [5], field-effect transistors (FETs) [5], surface acoustic wave (SAWs) [7], and quartz crystal microbalance (QCM) devices [8]. Oxide materials are most commonly used in resistive relative humidity sensors [5,9]. Many publications present studies of humidity sensing elements based on TiO_2_ [10,11,12,13,14], ZnO [15,16,17], SnO_2_ [18,19,20], ZrO_2_ [21,22], Fe_2_O_3_ [23,24,25], SiO_2_ [26,27,28], Al_2_O_3_ [29,30], spinel oxides [31], or perovskite oxides [32], etc. Oxide materials are mainly used due to their chemical and physical stability, high mechanical strength, easy realization of the sensor elements, good stability, and high sensitivity [33,34]. In a number of studies on *RH* sensors, nanostructured oxides are used due to their high surface-to-volume ratio [34,35]. Nanostructured materials are characterized by small grain size and large specific surface area, which leads to more surface-active sites and better sensitivity [22,36].

Among the basic oxide materials used for the elaboration of sensor elements, TiO_2_ stands out because of its beneficial properties, such as high hydrophilicity, chemical resistance, thermal stability, and mechanical strength [37,38,39]. These properties predetermine its suitability for efficient water vapor adsorption on the sensitive element surface, as evidenced by the distinguishable decrease in impedance of the respective sensors. The occurrence of highly developed porosity enhances the humidity sensor performance, because the appropriate size distribution facilitates the adsorption of water molecules on the surface of the respective sensing element [40,41].

Alloying with other materials improves the characteristics and parameters of humidity sensors. In this sense, especially the alkali metals, like Lithium [42,43,44], Potassium [45,46], and Sodium [10,47,48,49,50], have evinced their beneficial effect on the sensitivity, response and recovery times, and stability.

Specifically, the production of ceramic Na_4_Ti_5_O_12_-based sensors was reported in [47]. There, the contribution of Na-based additives for the improvement of sensor characteristics was investigated. The authors reported that the reached sensitivity range was about four orders of magnitude. The humidity sensitivity of Na- and Nb-doped TiO_2_-based ceramics was investigated in [51]. The studies in [10] present the combined influence of Bi_2_O_3_, PbO, and Na_2_CO_3_.10H_2_O on the characteristics and parameters of TiO_2_-based ceramic humidity sensors. The influence of sintering temperature on the humidity sensitivity of ceramics based on Na_1/3_Ca_1/3_Tb_1/3_Cu_3_Ti_4_O_12_ is presented in [48].

In the preparation of humidity sensing elements and other electronic elements, sol–gel technology is also used. In recent decades, the sol–gel route has enabled the development of entirely new generations of materials [52,53]. These include organically modified ceramics (ORMOCERs) [54,55] and advanced organic–inorganic hybrid materials [56,57], among them metal–organic framework (MOF)-based materials [58]. They have various applications, such as in the production of optoelectronic [57,58,59,60,61] and electrochromic [62] devices. Another newly developed generation of materials via sol–gel technology is the so-called “ormolites” [63,64]. These materials are well described in several extended review papers [58,65,66,67,68]. Moreover, the sol–gel route allows easy control over their composition, structure, and morphology by varying the synthesis conditions and the organometallic precursors used [69,70,71,72]. This approach also enables the synthesis of mixed oxide materials with beneficial properties, such as the advanced catalysts developed by Salinas et al. [73].

Essentially, the sol–gel method applied to organometallic compounds (i.e., metal alkoxides) involves the hydrolysis of these compounds in strongly acidic or alkaline media. This is followed by subsequent polymerization of the obtained free radicals (i.e., moieties), resulting in the formation of a polymer matrix. In this sense, it was demonstrated that the diammonium hexanitrocerate (NH_4_)_2_Ce(NO_3_)_6_ solutions possess strongly acidic properties [74]. In addition, it was established that these solutions keep their low pH even after significant NaOH additions, despite the appearance of Ce(OH)_4_ precipitate. These facts have inspired the idea to use this Ce-compound for hydrolysis/polymerization of titanium (IV) n-butoxide (TBOT) for elaboration of humidity sensors, as described in previous work [75].

An additional reason for selecting this complex cerium salt (i.e., diammonium hexanitrocerate) arises from its catalytic [76,77] and specific oxidant [78] properties. Consequently, its addition to the initial sols should favor the overall gel formation process.

Finally, the following inferences can be drawn, summarizing the statements, mentioned above: (i) humidity sensors possess a great importance for the human life and activities; (ii) TiO_2_ provides superior chemical and physical properties, including stability and high sensitivity of humidity sensors; (iii) nanomaterials enhance the sensitivity to relative humidity (*RH*); (iv) alkali metals, such as Na, contribute to higher sensitivity, shorter response and recovery times, and better stability; (v) humidity sensors based on sodium-doped TiO_2_ are, to our knowledge, mainly fabricated using ceramic technology; (vi) the involvement of Ce compounds has a beneficial effects on gel formation; (vii) the sol–gel method enables the synthesis of entirely new generations of materials, with easy control of their composition, structure and morphology.

Summarizing these facts has led to the concept of elaborating humidity-sensing elements based on TiO_2_, doped with Na in the presence of Ce ions, prepared via the sol–gel method, and sintered at 400 °C and 800 °C. The present research therefore aims to perform a comparative study on the influence of two Na-providers—sodium hydroxide (NaOH) and sodium tert-butoxide ((CH_3_)_3_CONa)—on the characteristics and parameters of the sensing elements obtained by the sol–gel method, as well as on the influence of sintering temperature. As a result of the described method and the materials, sensor elements with improved humidity sensing properties were obtained compared to the reference samples prepared using the same technology and sintering temperatures but without Na additives.

## 2. Materials and Methods

### 2.1. Materials and Sample Preparation

Several precursors were used for the sol–gel layer preparation and deposition in the present study. Each one of them has its own function in the entire sol–gel layer formation process, as follows:

- Titanium (IV) n-butoxide with CAS 5593-70-4, product of Thermo Fisher Scientific Inc.—Waltham, MA, USA with purity grade above 99%. It was used as precursor for the basic TiO2 matrix formation.
- Isobuthanol, with CAS: 78-83-1, produced by Thermo Fisher Scientific Inc.—Waltham, MA, USA with a purity grade higher than 99%. This compound was used for dissolving of all rest sol–gel system ingredients.
- Diammonium hexanitrocerate with CAS 16774-21-3, produced by Fluka Chemie GmbH—Buchs, Switzerland. It is sold under the commercial name “Ceric ammonium nitrate”, with 99% purity. It serves simultaneously as hydrolysis initiator and source of Ce4+-ions. This compound supplies rather strong acidic conditions during the sol–gel synthesis and provides CeO2 content in the final layers after sintering.
- Sodium hydroxide with CAS 1310-73-2, produced by Merck KGaA—Darmstadt, Germany with 98–99% of purity. The determination of the impacts of its addition to the primary sol is directly related to the object of the present study.
- Sodium tert-butoxide with CAS 865-48-5, produced by Alfa Aesar GmbH—Karlsruhe, Germany, with 97% purity grade. The definition of the effects of its addition to the primary sol is also directly related to the object of the present study.

The development of the sensing elements was divided into two stages: preparation of the sol–gel systems and deposition of the layers with subsequent final sintering.

- Preparation of the sol–gel systems

Initially, a saturated solution of diammonium hexanitratocerate ((NH_4_)_2_Ce(NO_3_)_6_) in isobutanol (C_4_H_9_OH), with an approximate content of 11.51 g.dm^−3^ was prepared by stirring for 45 min. It was left in the refrigerator overnight. Three portions of 50 mL of this solution were poured into three beakers the next day. The first one was left without an additive, whereas two different sodium compounds were added to the second and the third portions, reaching a ratio of 0.5 g additive to 50 mL of the solution, respectively. Thus, three mixtures were acquired, as follows:

(1) Without Na precursor (reference);
(2) Inorganic additive—sodium hydroxide (NaOH);
(3) Organometallic additive—sodium tert-butoxide ((CH3)3CONa).

All additions were carried out at room temperature and with intensive stirring for 45 min. The resulting solutions were left again overnight in the refrigerator. These two additives were selected for the present research, since the former (i.e., NaOH) is the simplest and most widely used alkaline inorganic compound. The latter ((CH_3_)_3_CONa) is an organometallic compound with similar organic moieties to those of the main Ti-compound and the organic solvent (i.e., isobutanol) used for the initial sol preparation. In addition, both these Na-compounds are strongly alkaline. Their alkalinity causes a partial Ce(OH)_4_ precipitation due to their interaction with the dissolved (NH_4_)_2_Ce(NO_3_)_6_. Further, the Ce(OH)_4_ precipitate converts to CeO_2_ during the subsequent thermal treatment, described below.

Sol preparation was carried out by adding titanium (IV) n-butoxide (C_16_H_36_O_4_Ti) to the indicated solutions in a volume ratio of 1:1 (i.e., 10 mL of one of the above-described solutions to 10 mL of titanium (IV) n-butoxide—TBOT).

This proportion has empirically shown to be suitable. Indeed, on one hand, it provides a sufficiently low concentration of the precursors to prevent premature gelation during the layer deposition. On the other hand, the precursor concentrations should be high enough to form distinguishable adsorbing layers with a beneficial effect on the sensor properties.

- Film deposition and sintering

Prior to the layer formation, the deposition solutions described above were heated at 85 °C for 30 min. The layer depositions were performed on corundum substrates with silver-palladium electrodes, well described in previous work [13]. Their surfaces were activated by immersion in an acetone–ether mixture (1:1). The depositions were performed in a triple repetition of 30 min immersion in the solutions, followed by 30 min drying at the same temperature. The final gelation of the films proceeded during their drying at ambient conditions for at least overnight.

Meanwhile, the residual parts of each of the three sols were poured into Petri dishes for subsequent sintering of powder materials for XRD analysis.

Samples with the formed gel films, together with the materials in the Petri dishes, were sintered for 30 min at 400 °C or at 800 °C, respectively.

The samples thus obtained, and the corresponding powder materials, were designated as follows: reference samples—TC_REF_xxx; samples with the addition of NaOH: TC_NOH_xxx; samples with the addition of sodium tert-butoxide: TC_STB_xxx. The sign “xxx” is replaced by the corresponding sintering temperature: 400 °C or 800 °C.

### 2.2. Surface Morphology and Structural Characterization

The morphological features of the studied samples were observed at high resolution, corresponding to a 5000-fold magnification. The observations were performed by scanning electron microscopy (SEM), using TESCAN, SEM/FIB LYRA I XMU. It was supported by an additional Quantax 200 detector produced by BRUKER for additional chemical element analysis by energy-dispersive spectroscopy (EDX). It enabled acquisition of map data images of the surface layers.

In addition, powders obtained from the respective gels and sintered at 400 °C and 800 °C were analyzed by X-ray diffractometry (XRD). For this purpose, a Philips PW 1050 diffractometer equipped with a CuKα X-ray emitter and a monochromator was used. The measurements were carried out in the 2*θ* range from 7° to 95°, with a step size of 0.05° 2*θ*.

### 2.3. Electrical Measurements

The response of the humidity sensing elements was evaluated by measuring resistance (*R*), capacitance (*C*), impedance (*Z*), and phase angle (*θ*) as relative humidity varied at a fixed temperature. Data were collected using a Precision LCR Meter MIFA (Zurich Instruments AG, Zurich, Switzerland) with an accuracy of 0.05%. Measurements were conducted at a sinusoidal excitation signal of 500 mV and a frequency of 20 Hz. Humidity and temperature around the sensors were regulated using a chamber controlled by the HygroGen2-XL (Rotronic Instruments Ltd., Bassersdorf, Switzerland). This system maintained relative humidity between 5% and 95% with an accuracy of 0.1% *RH*, and temperatures from 0 °C to 60 °C with a maximum deviation of 0.01 °C.

## 3. Results and Discussion

### 3.1. SEM and EDX Analyses

**Figure 1:**
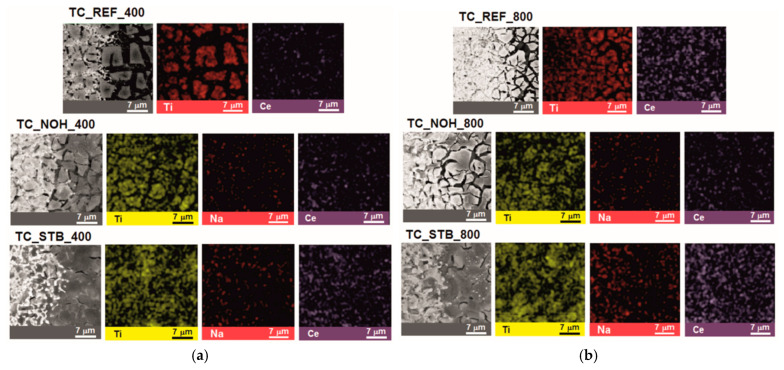
SEM and EDX images of the surface layers of samples TC_REF, TC_NOH, and TC_STB, sintered at temperatures: (**a**) 400 °C; and (**b**) 800 °C.

SEM and EDX images of the surface layers of the studied samples TC_REF, TC_NOH, and TC_STB, sintered at temperatures of 400 °C and 800 °C, are presented in Figure 1.

SEM images show that at both sintering temperatures (400 °C and 800 °C), the surface layers contain aggregates of the sintered material with channel-like spaces between them, most pronounced in samples TC_REF and TC_NOH. EDX map analysis shows the presence of Ti and Ce in the aggregates of the sintered surface material for the reference sample TC_REF, and for samples TC_NOH and TC_STB—the presence of Ti, Na, and Ce in the sintered surface layers.

For the samples with organometallic additive TC_STB, the deposited thin films fill the spaces between the grains of the ceramic substrate, and the channel-like spaces are very small.

The distinguishable morphologies of the layers, prepared with the addition of sodium tert-butoxide, might be a consequence of reactions between this compound and the organic moieties of the other precursors during the sol–gel process, as described in [79,80]. Such reactions should result in the redistribution of Na, Ce, and Ti, thereby affecting the final morphology of the sintered layers.

### 3.2. XRD Analysis

**Figure 2:**
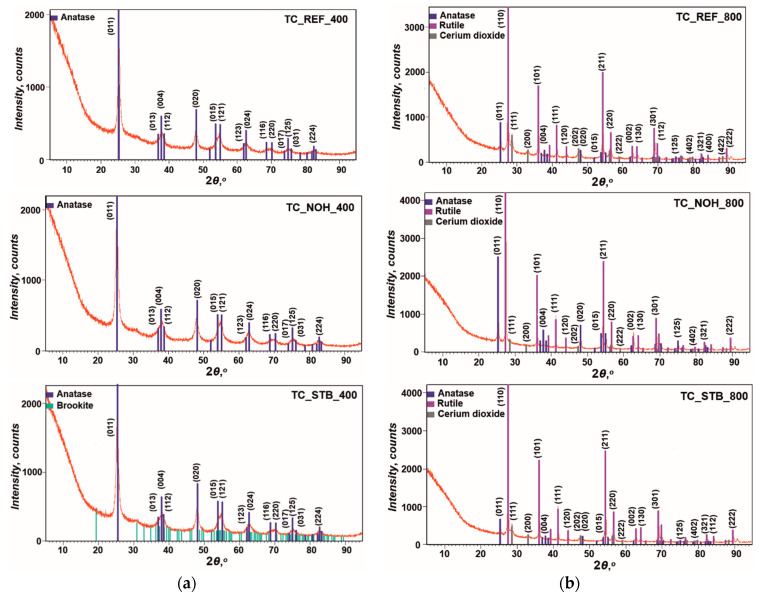
XRD images of the material of samples TC_REF, TC_NOH, and TC_STB, sintered at temperatures: (**a**) 400 °C; and (**b**) 800 °C.

The XRD patterns for the material of the studied samples TC_REF, TC_NOH, and TC_STB, for each of the sintering temperatures are presented in Figure 2.

At a sintering temperature of 400 °C for all samples TC_REF, TC_NOH, and TC_STB, the structure of the material of the surface layers corresponds to TiO_2_—anatase (Crystallography Open Database—COD, № 96-900-9087). The XRD image for sample TC_STB_400 shows very small diffraction peaks of brookite, with the strongest peak being in the amorphous halo with a low height. Therefore, it can be assumed that its amount is insignificant. Further, crystalline phases were not detected for cerium or for sodium at this sintering temperature. This fact is to be expected, since research on similar systems has shown that the CeO_2_ occurs in a vitreous state at temperatures below 500 °C [81], and crystallizes at 800 °C [82]. Other authors [83] report synthesis of Na_2_O-TiO_2_ glasses at 500 °C and 600 °C, respectively. Consequently, the entire sodium oxide content occurs in an amorphous state.

At the higher sintering temperature of 800 °C, a process of transformation of the TiO_2_ structure from anatase to rutile (COD, № 96-900-9084), according to [84], with a predominant presence of anatase occurred. In addition, for all samples sintered at 800 °C, the presence of crystalline cerium dioxide—CeO_2_ (COD, № 96-900-9009) was detected, confirming the statements in [82]. However, sodium-containing crystalline phases were not detected. This is probably because an amorphous Na_2_O-TiO_2_ phase remains after sintering at this temperature, comprising the entire Na content.

Based on the Scherrer equation [85], the crystallite sizes of anatase, rutile, and crystalline CeO_2_ were calculated from the obtained XRD data. The following crystallite sizes were determined:

- For samples, sintered at 400 °C: TC_REF_400—anatase 12.3 nm; TC_NOH_400—anatase 9.5 nm; TC_STB_400—anatase 9.9 nm;
- For samples, sintered at 800 °C: TC_REF_800—anatase 31.8 nm, rutile 69.1 nm, and cerium dioxide 56.1 nm; TC_NOH_800—anatase 53.2 nm, rutile 93.8 nm, and cerium dioxide 43.9 nm; TC_STB_800—anatase 132.4 nm, rutile 89.8 nm, and cerium dioxide 25.7 nm.

It can be noted that at the lower sintering temperature of 400 °C, the crystallite sizes, in this case of anatase, are significantly smaller than those of anatase in the samples sintered at 800 °C. In addition, the crystallite sizes of rutile and CeO_2_ at 800 °C are also larger than those of anatase at 400 °C. In principle, larger crystallite sizes also result in larger intercrystalline spaces.

The filling of the smaller spaces in the structure starts at lower levels of relative humidity. Therefore, higher sensitivity at lower levels of relative humidity is expected for the samples sintered at 400 °C, and better linearity of the characteristics on a semi-logarithmic scale. Larger intercrystalline spaces in the samples sintered at 800 °C will lead to the manifestation of sensitivity at higher levels of relative humidity. In addition, the adsorbed moisture will be released more slowly from these larger intercrystalline spaces when it returns to the air during desorption processes.

Thus, the material structure of the surface layers will influence the sensory characteristics of the developed elements.

### 3.3. Electrical Characteristics

**Figure 3:**
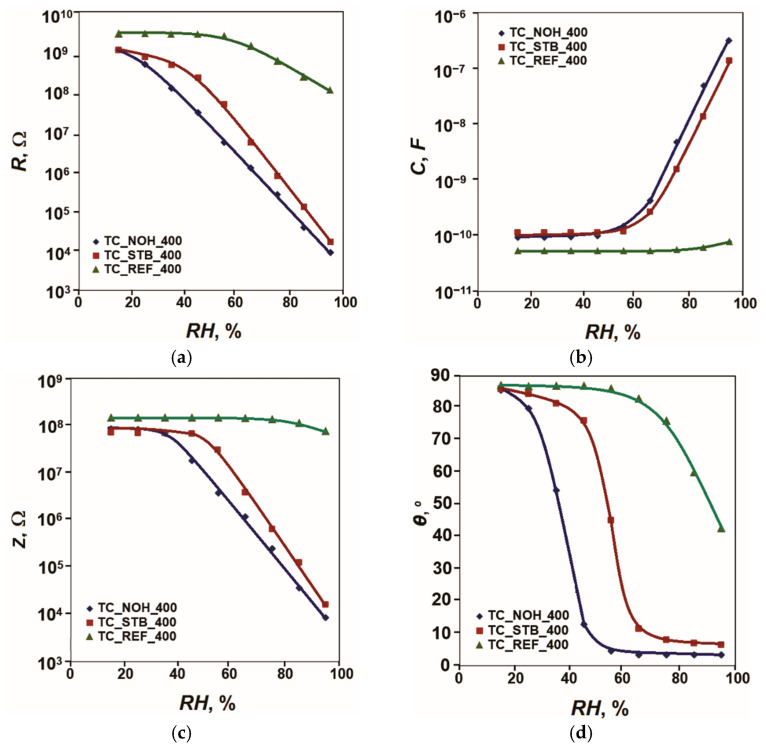
Characteristics: (**a**) *R* = *f*(*RH*), (**b**) *C* = *f*(*RH*), (**c**) *Z* = *f(RH*); and (**d**) *θ* = *f(RH*) for samples TC_REF, TC_NOH and TC_STB, sintered at 400 °C, studied at 20 Hz and 25 °C.

All the studied sensors underwent analyses of their sensitivity in the conditions described in the experimental part. The acquired sensor characteristics of resistance *R*, capacitance *C*, impedance *Z*, and phase angle *θ* versus relative humidity in the range (15–95)% *RH* at 20 Hz and 25 °C for the samples, sintered at 400 °C, are plotted in Figure 3.

Of the studied parameters *R*, *C,* and *Z*, the resistance *R* has the largest relative variation in the range of relative humidity variation from 15% *RH* to 95% *RH* and is the most informative of these parameters. The maximum relative change (*R_max_*/*R_min_*) of the parameter *R* of the humidity sensing elements in the range from *RH_min_* to *RH_max_* can be considered as the maximum relative sensitivity:

(1) $$S_{Rrel\;max}=\;R_{max}/R_{min}$$

in terms of resistance over the entire range of relative humidity.

Since the change in resistance *R* is very large, the sensing response in orders of magnitude can be calculated as follows:

(2) $$L_{R}=lg\left(R_{max}/R_{min}\right)=lg\left(R_{max}\right)-lg\left(R_{min}\right)$$

For the reference sample TC_REF_400, this change is about 1.4 orders of magnitude. The inclusion of Na as an alloying additive increases the sensitivity of the sensor elements and the range of variation in R in accordance with Figure 3. This change reaches 5.1 orders of magnitude for sample TC_NOH_400 and 4.8 orders of magnitude for sample TC_STB_400. The presence of more pronounced channel-like spaces in the structure of sample TC_NOH_400 compared to those in sample TC_STB_400 facilitates the penetration of moisture into the aggregate material. This is expressed in the relatively higher sensitivity of sample TC_NOH_400. It should also be noted that of the studied samples doped with Na and sintered at 400 °C, sample TC_NOH_400 has a relatively well-expressed linear characteristic in semi-logarithmic scale, characteristic *R* = *f*(*RH*).

The change in the phase angle *θ* for the reference sample TC_REF_400 is 44° (from 87° to 43°), while for the samples doped with Na, it increases, reaching 82° (from 86° to 4°) for the sample TC_NOH_400 and 79° (from 86° to 7°). For the sample TC_NOH_400, this change is up to about 55% *RH*, and for the sample TC_STB_400—up to 65% *RH*, after which the change in *θ* is insignificant.

**Figure 4:**
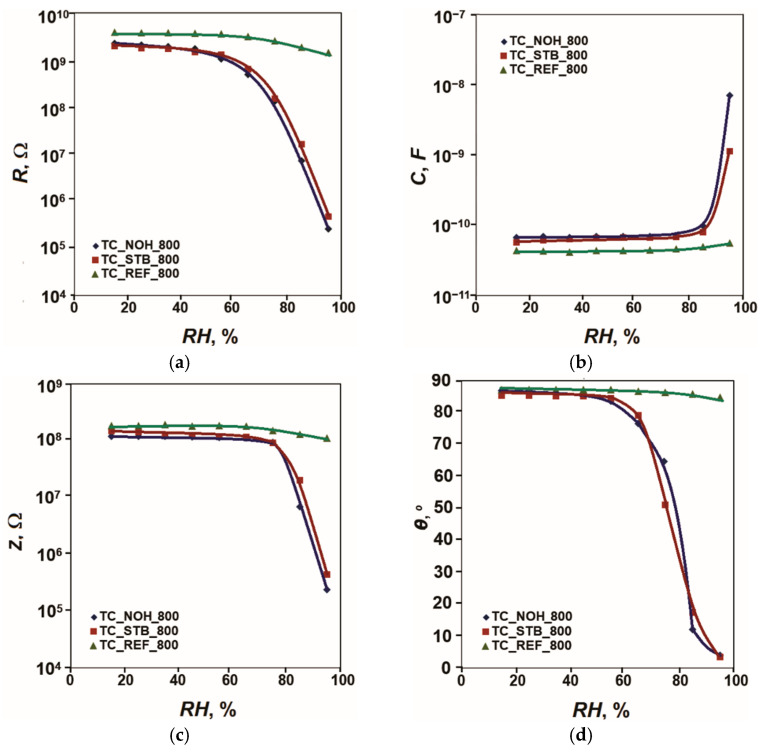
Characteristics: (**a**) *R* = *f*(*RH*), (**b**) *C* = *f*(*RH*), (**c**) *Z* = *f*(*RH*); and (**d**) *θ* = *f*(*RH*) for samples TC_REF, TC_NOH and TC_STB, sintered at 800 °C, studied at 20 Hz and 25 °C.

The acquired sensor characteristics of resistance *R*, capacitance *C*, impedance *Z* and phase angle *θ* to relative humidity in the range (15–95)% *RH* at 20 Hz and 25 °C for the samples, sintered at 800 °C, are presented in Figure 4.

For the reference sample TC_REF_800, the change in resistance *R* is less than one order of magnitude (0.4). For the samples doped with Na, this change reaches 4.0 orders of magnitude for the sample TC_NOH_800. For the sample TC_STB_800, it reaches 3.6 orders of magnitude. Sample TC_NOH_800 shows higher sensitivity than TC_STB_800, as in the samples sintered at 400 °C. This difference can be attributed to the more pronounced channel-like spaces between the aggregates in the sample with NaOH addition. The change in phase angle *θ* for the reference sample TC_REF_800 is 2° (from 87° to 85°). For the samples doped with Na, this change increases. For the sample TC_NOH_800, it reaches 83° (from 87° to 4°), and for the sample TC_STB_800, it reaches 81° (85° to 4°).

The data for the sensitivity of the investigated samples after sintering at 400 °C or 800 °C, acquired for the rom 15% *RH* to 95% *RH,* are summarized in Table 1.

Compared to the reference samples, the presence of Na as an additive for both Na-sources leads to an increase in the range of changes in their characteristics, *R* = *f*(*RH*). For the samples sintered at 400 °C, the increase in their sensitivity range is 3.7 orders of magnitude (up to 5.1 orders of magnitude) for the TC_NOH_400 sample and 3.4 orders of magnitude (up to 4.8 orders of magnitude) for the TC_STB_400 sample. For the samples sintered at 800 °C, this increase in sensitivity is 3.6 orders of magnitude (up to 4.0 orders of magnitude) for the TC_NOH_800 sample and 3.2 orders of magnitude (up to 3.6 orders of magnitude) for the TC_STB_800 sample.

In a previous work [75], it was established that the occurrence of Ce in the humidity sensing layers obviously increases the sensitivity (especially at 400 °C), in comparison to those prepared with pure TiO_2_. The present study has revealed that the beneficial effect of Na-additive on the sensitivity of the resulting films is remarkable, compared to the humidity sensitivity of the corresponding reference samples based on TiO_2_ with Ce doping. This effect is significant in both cases, when the inorganic (NaOH) and the organometallic ((CH_3_)_3_CONa) sodium compounds are used. The results obtained convincingly show that the addition of Na results in a rather high sensitivity of the layers, sintered at each respective temperature (i.e., 400 °C and 800 °C).

When comparing the characteristics and parameters of the samples with Na additives at both sintering temperatures, it was found that the samples sintered at 400 °C have a higher sensitivity compared to those sintered at 800 °C.

The material of the samples sintered at 400 °C mainly contains anatase. This phase is characterized by higher hygroscopicity compared to rutile [86]. In the material of the samples sintered at 800 °C, in accordance with the XRD images, a transition of anatase to rutile with lower hygroscopicity has occurred. In addition, as mentioned above, the crystallite sizes of anatase, rutile, and CeO_2_ in the samples sintered at 800 °C are larger compared to the size of anatase crystallites at 400 °C. Therefore, the filling of the intercrystalline spaces in samples sintered at 400 °C begins at lower levels of relative humidity. This is confirmed by the nature of the obtained sensor characteristics, presented in Figure 3 and Figure 4. Regarding the surface morphology, the samples TC_NOH exhibit more pronounced and wider channel-like spaces between the aggregates in the surface layers, compared to the samples TC_STB. These structural features facilitate easier moisture penetration into the aggregate material. As a result, the samples prepared using sodium hydroxide show relatively higher sensitivity than those prepared with sodium tert-butoxide at the same sintering temperatures. The best linearity on a semi-logarithmic scale is observed for sample TC_NOH_400.

The samples studied are oxide-type humidity sensing elements. Regarding their sensitivity mechanism, these sensors undergo sequential chemical and physical adsorption processes. These are followed by capillary condensation of water vapor. Initially, at low levels of relative humidity, chemical adsorption of water vapor occurs. This leads to the adsorption of hydroxyl ions [87,88,89,90,91] and an increase in the material’s conductivity. In the prepared samples, chemical adsorption is governed by the presence of Ti and Ce ions in the surface layers of the reference samples. In the samples with Na additives, it is governed by Ti, Na, and Ce ions in the surface layers. This results in the formation of hydroxyl groups X-OH. As humidity increases, physical adsorption and protonic (H+ ions) conductivity, based on the Grotthuss mechanism [88,89], become significant. As a result, a notable increase in conductivity and a corresponding decrease in resistance are observed. At high levels of relative humidity, capillary condensation processes also begin. This occurs in the presence of pores and, in this case, also of channel-like spaces between the aggregates in the surface layers. These processes are based on Kelvin’s equation [90,91].

This mechanism of sensor sensitivity corresponds to the nature of the change in the obtained sensor characteristics *R* = *f*(*RH*) as relative humidity increases. When comparing these characteristics, it is found that for samples with Na additives, their resistance decreases significantly compared to that of the reference samples with increasing humidity *RH*. This decrease is most significant for relative humidity levels above 20% *RH* for sample TC_NOH_400, above 30% *RH* for sample TC_STB_400, and above 50% *RH* for samples TC_NOH_800 and TC_NOH_800, when the physical adsorption processes begin, followed by capillary condensation.

For the samples studied, the hysteresis of their characteristics *R* = *f*(*RH*) was also determined by measuring their resistance at different levels of humidity, when it increases from 15% *RH* to 95% *RH*, and then in the opposite direction. Numerically, the hysteresis *F* is calculated as the ratio of the maximum difference $∆R_{max}$ between the measured resistance values when the relative humidity changes in both directions, to the range of resistance change $R_{FS}=\;R_{max}-\;R_{min}$, expressed in percent as follows:

(3) $$F=\left(∆R_{max}/R_{FS}\right)\times 100\%$$

**Figure 5:**
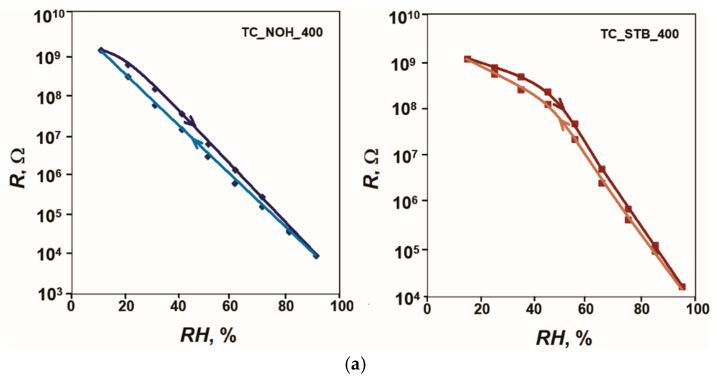

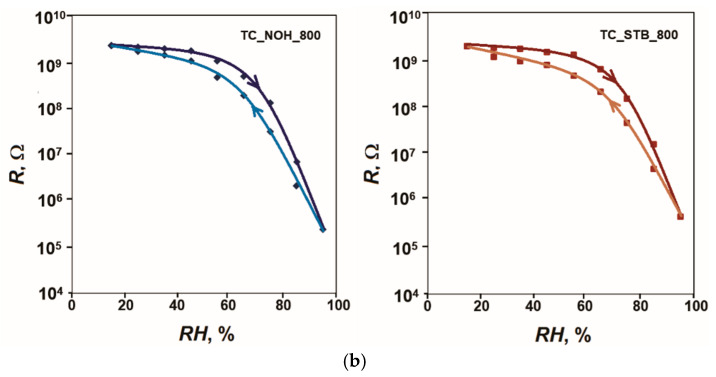
Hysteresis of the characteristics *R* = *f(RH*) of the samples: (**a**) TC_NOH and TC_STB, sintered at 400 °C, and (**b**) TC_NOH and TC_STB, sintered at 800 °C, studied at 20 Hz and 25 °C.

Five measurement cycles were performed for each sample. The results showed very good repeatability of the characteristics, and the largest difference in the readings in the ascending and descending sequence in the respective cycles was taken for calculations. Figure 5 shows the hysteresis of the characteristics *R* = *f(RH*) of the samples TC_NOH and TC_STB, sintered at 400 °C and at 800 °C, respectively, for humidity adsorption and desorption at 20 Hz and 25 °C. Based on the obtained results, the following values for the hysteresis *F* of the developed samples were obtained: 3.8% for sample TC_NOH_400, 2.9% for sample TC_STB_400, 4.7% for sample TC_NOH_800, and 6.2% for sample TC_STB_800. For samples sintered at 400 °C, the difference in hysteresis was small. For sample TC_STB_400, the sensitivity was relatively less pronounced compared to that of sample TC_NOH_400. Therefore, the relatively smaller amount of adsorbed moisture is returned to the air faster.

For samples sintered at 800 °C, the hysteresis increased compared to samples sintered at 400 °C. As previously stated, the larger intercrystalline spaces in samples sintered at 800 °C slowed the release of moisture from these spaces, resulting in greater hysteresis for these samples. The narrower, channel-like spaces in the surface structure of sample TC_STB_800, compared to TC_NOH_800, further retarded moisture release to the atmosphere. Therefore, sample TC_STB_800 exhibits the greatest hysteresis.

The determination of the response characteristics of the developed samples was carried out when switching the relative humidity from a low level of 15% to a high level of 95%. The recovery characteristics were determined by switching back from the specified high to low level of relative humidity.

The response time for adsorption and recovery time for desorption were determined as the time to reach 90% of the total change in the respective resistance during each switching process.

**Figure 6:**
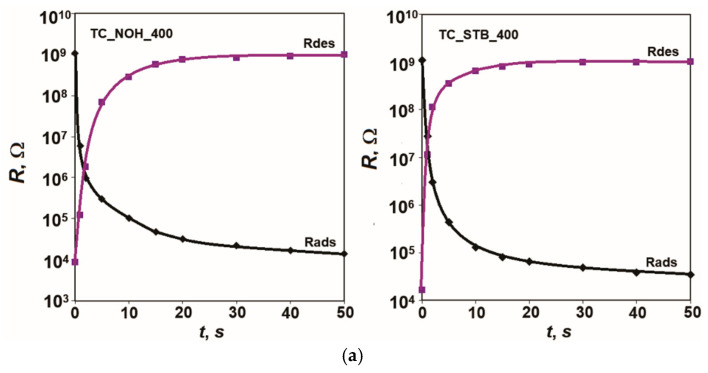

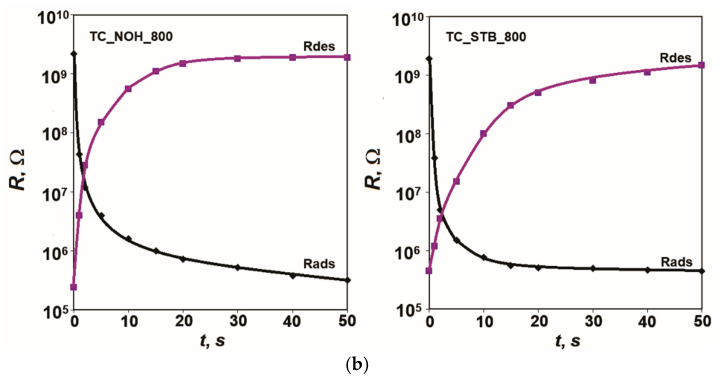
Response and recovery characteristics *R* = *f*(*t*) of samples: (**a**) TC_NOH and TC_STB, sintered at 400 °C, and (**b**) TC_NOH and TC_STB, sintered at 800 °C, studied at 20 Hz and 25 °C.

The obtained response and recovery characteristics when switching between the specified relative humidity levels at 20 Hz and 25 °C are presented in Figure 6.

The response and recovery times were determined based on five switching cycles for each sample. The results showed very good repeatability in the individual measurements and reliability of the assessments.

According to the obtained results, fast moisture adsorption for all studied samples doped with Na (TC_NOH_400, TC_STB_400, TC_NOH_800, TC_STB_800) is observed. This is due to the presence of Ti, Na, and Ce ions and the described sensor sensitivity mechanism. The response time, *t_ads_*, for all developed samples with Na additives, is very short—up to 2 s.

From the recovery characteristics, recovery times for the studied samples were determined: *t_des_*_TC_NOH_400_ = 18 s, *t_des_*_TC_STB _400_ = 14 s, *t_des_*_TC_NOH_800_ = 20 s, and *t_des_*_TC_ STB_800_ = 40 s. The samples sintered at 400 °C have a relatively shorter desorption time. For samples sintered at 800 °C, the recovery time relatively increases compared to that of the corresponding samples sintered at 400 °C.

Similarly to the effects described for hysteresis, the larger intercrystalline spaces in the samples sintered at 800 °C lead to a slower desorption process and a longer recovery time, *t_des_*. For the TC_STB_800 sample, the channel-like spaces are smaller than those in TC_NOH_800, which has an additional impact. In this case, the recovery time is the longest.

In addition to all the described repeated measurements for the samples studied, measurements were performed on five samples from each group to check the repeatability of the sensor elements’ characteristics and parameters. Statistical processing of the results was performed. The deviations in the corresponding parameters for each series do not exceed ±6%, which confirms good repeatability of the results.

The characteristics and parameters of these samples were also measured after an interval of 9 months. This approach helped monitor temporal stability and aging effects. After statistical processing, a deviation of about ±7% was obtained, indicating that the samples remained stable.

A comparison of the main parameters of the developed sensor elements (sample TC_NOH_400) and other humidity sensor elements with Na additives from recent literature is presented in Table 2.

Most of the humidity sensing elements with titania, titanates, and Ti composites, doped by Na additives, from our known literature, were made using ceramic technology. The proposed sensor elements were prepared using simple sol–gel technology. The sensitivity of the developed sensor elements is high, with sensing response for resistance reaching over five orders of magnitude. The response time is shorter—2 s, the recovery time is 18 s, and less than that of most of the considered sensor elements. The hysteresis 3.8% is comparable to or smaller than that of the given data for other sensors.

The use of sol–gel technology provides a number of advantages. As mentioned in the Introduction, this technology enables the fabrication of nanostructured layers. This leads to enhanced sensitivity of the sensing elements due to the larger specific surface area available for moisture adsorption. Thin sol–gel layers allow water molecules to penetrate the material and subsequently return to the air more quickly, resulting in shorter response and recovery times. The sol–gel approach also allows easy adjustment of sensor dimensions by varying the size of the substrate on which the layer is deposited. Moreover, these dimensions can be smaller than those of ceramic samples.

The fabrication process does not require complex equipment. In addition, the precursors used in sol–gel methods are most usually easily accessible and allow simple variation in order to achieve positive effects on the properties of the elements. Samples prepared via the sol–gel method can be sintered at lower temperatures compared to ceramic ones. This not only facilitates sintering but also requires lower energy consumption. Furthermore, the materials used in this technology are compatible with the microelectronic industry, enabling the production of not only compact but also integrated sensors.

However, to realize the above-mentioned advantages, a key aspect in the development of sol–gel-based sensors is the selection of appropriate precursors, fabrication conditions, and sintering regimes. The humidity sensing elements proposed in this work are characterized by high sensitivity, short response and recovery times, good repeatability of characteristics, and long-term stability. These properties can be achieved using the described technology and sintering at a relatively low temperature of 400 °C. High sensitivity over a wide humidity range, along with a fast response, is a prerequisite for their use in humidity measurement and control, including in environments with rapidly changing relative humidity.

The demonstrated advantages of the developed humidity sensor elements confirm the positive effect of using TiO_2_ alloying with Na additives in the presence of Ce ions, combined with the sol–gel approach. The excellent humidity-sensing properties make these elements suitable for a wide range of practical applications.

## 4. Conclusions

Humidity-sensing elements based on Na-doped TiO_2_ in the presence of Ce ions were developed. They were prepared via a sol–gel method and sintered at 400 °C and 800 °C. The influence of Na precursors—inorganic (sodium hydroxide) and organometallic (sodium tert-butoxide)—on the characteristics and parameters of the sensing elements was studied.

The applied analyses comprised the following: (i) the sample morphology (by scanning electron microscopy (SEM)); (ii) chemical composition (by energy dispersion X-ray spectroscopy (EDX)); (iii) structure (by X-ray diffractometry); (iv) humidity sensing properties, by impedance spectroscopy, applied to the samples exposed to exact humidity rates.

SEM observations showed that, at both sintering temperatures (400 °C and 800 °C), the surface layers contain aggregates of sintered material with channel-like spaces between them. The only exclusion belongs to the samples, prepared by the addition of sodium tert-butoxide. In these samples, the deposited thin films fill the spaces between the grains of the ceramic substrate, and the channel-like spaces are very narrow. This difference is probably due to reactions between this compound and the organic moieties of the rest precursors, during the sol–gel process.

The EDX analyses have revealed a relatively uniform distribution of the main elements, composing the layers.

The X-ray diffraction (XRD) patterns of all samples sintered at 400 °C indicate the presence of the anatase phase. No crystalline phases were detected for cerium or sodium, as the oxides of these elements remain amorphous after sintering at this temperature.

The XRD results for the layers sintered at 800 °C reveal the occurrence of anatase, rutile, and crystalline CeO_2_, and only Na remains in the amorphous oxide phase.

Samples synthesized with sodium (Na) additive precursors demonstrate higher sensitivity than the corresponding reference samples.

All synthesized samples exhibit high sensitivity, ranging from 3.6 to 5.1 orders of magnitude. These samples demonstrate low hysteresis values between 2.9% and 6.2%, short response times of up to 2 s, and recovery times between 14 and 40 s. The highest sensitivity is observed in samples TC_NOH_400 and TC_STB_400, sintered at 400 °C, with resistance changes reaching up to over 5 orders of magnitude. Samples sintered at 800 °C (TC_NOH_800 and TC_STB_800) achieve resistance changes up to four orders of magnitude. Furthermore, samples sintered at 400 °C display lower hysteresis and shorter recovery times compared to those sintered at 800 °C, while maintaining similar response times. Sample TC_NOH_400 demonstrates the highest sensitivity (5.1 orders of magnitude) and the most linear response on a semi-logarithmic scale. The measurements indicate a high degree of data repeatability and sensor stability.

The proposed humidity sensor elements based on titania, doped with Na in the presence of Ce ions, offer high humidity sensing performances, use inexpensive materials, and employ simple manufacturing technology, making them effective for practical applications. These highly sensitive elements can be used both in portable devices and in systems for measuring, controlling, and monitoring humidity in various fields.

## Figures and Tables

**Table 1:** Comparison of the sensitivity data for (15–95)% *RH* acquired from the investigated sensors.

Sample	*R_max_*, Ω	*R_min_*, Ω	*S_Rrel max_*, -	Sensing Response *L_R_*, Orders of Magnitude
TC_REF_400	2.73 × 10^9^	1.05 × 10^8^	2.59 × 10^1^	1.4
TC_NOH_400	1.08 × 10^9^	8.69 × 10^3^	1.24 × 10^5^	5.1
TC_STB_400	1.09 × 10^9^	1.64 × 10^4^	6.63 × 10^4^	4.8
TC_REF_800	3.81 × 10^9^	1.41 × 10^9^	2.70 × 10^0^	0.4
TC_NOH_800	2.20 × 10^9^	2.41 × 10^5^	9.12 × 10^3^	4.0
TC_STB_800	1.90 × 10^9^	4.43 × 10^5^	4.29 × 10^3^	3.6

**Table 2:** Comparison of the main parameters of the developed humidity sensing elements and other humidity sensors.

Sensing Material	*RH* Range, %	Sensing Response, Orders of Magnitude	Response Time/ Recovery Time, s	Hysteresis, %	Reference
(Na_0_._5_Bi_0_._5_)_0_._94_Ba_0_._06_TiO_3_ Ceramic	15–90	3	68/125	4.69	[92]
TiO_2_/(K_0_._5_Na_0_._5_)NbO_3_ Nanocomposite	12–94	5	25/38	<5	[93]
Na_4_Ti_5_O_12_ Ceramic	11–75	4	34/199	4.15	[47]
Na_0_._5_Bi_0_._5_TiO_3_ Ceramic	11–94	5	43/87	13.5	[94]
Na_0_._5_Bi_x_TiO_3_ Ceramic	11–75	5	4.4/29.5	<10	[49]
NaTaO_3_/TiO_2_ Hot solvent method	11–95	4.5	13/9	8.5	[95]
Bi_0_._5_Na_0_._5_TiO_3_–Bi_0_._5_K_0_._5_TiO_3_ Metal–organic decomposition method	11–95	4	20/60	∼4	[96]
Ti-Na-Ce-O Sol–gel method, surface layers	15–95	5	2/18	3.8	this work

## Data Availability

The data from this study are presented in the article; further inquiries can be directed to the corresponding author.

## References

[1] Lee C.-Y., Lee G.-B. (2005). Humidity sensors: A review. Sens. Lett..

[2] Wernecke R., Wernecke J. (2014). Industrial Moisture and Humidity Measurement: A Practical Guide.

[3] Ku C.-A., Chung C.-K. (2023). Advances in Humidity Nanosensors and Their Application: Review. Sensors.

[4] Faharani H., Wagiran W., Hamida M.N. (2014). Humidity sensors principle, mechanism and fabrication technologies: A comprehensive review. Sensors.

[5] Blank T.A., Eksperiandova L.P., Belikov K.N. (2016). Recent trends of ceramic humidity sensors development: A review. Sens. Actuators B Chem..

[6] Korotcenkov G. (2021). Handbook of Humidity Measurement, Volume 2: Electronic and Electrical Humidity Sensors.

[7] Han Y.C., Kong X.Y., Wu W., Li J.S., Yang X., Guo Y.J., Fu Y.Q., Torun H., Xiang X., Tang Y.L. (2022). Environment-friendly surface acoustic wave humidity sensor with sodium alginate sensing layer. Micro Nano Eng..

[8] Yao Y., Huang X., Chen Q., Zhang Z., Ling W. (2020). High Sensitivity and High Stability QCM Humidity Sensors Based on Polydopamine Coated Cellulose Nanocrystals/Graphene Oxide Nanocomposite. Nanomaterials.

[9] Korotcenkov G. (2020). Handbook of Humidity Measurement, Volume 3: Sensing Materials and Technologies.

[10] Nenov T., Nenova Z. (2013). Multi-Objective Optimization of the Parameters of TiO_2_-based Ceramic Humidity Sensors. Ceram. Int..

[11] Musa M.Z., Mamat M.H., Vasimalai N., Subki A.S.R.A., Hassan H., Malek M.F., Ahmad M.Y., Rusop M. (2022). Recent Progress on Titanium Dioxide-Based Humidity Sensor: Structural Modification, Doping, and Composite Approach. Enabling Industry 4.0 Through Advances in Manufacturing and Materials. Lecture Notes in Mechanical Engineering.

[12] Zahidi M.M., Mamat M.H., Malek M.F., Yaakob M.K., Ahmad M.K., Bakar S.A., Mohamed A., Subki A.S.R.A., Mahmood M.R. (2022). Evaluating Different TiO_2_ Nanoflower-Based Composites for Humidity Detection. Sensors.

[13] Kozhukharov S., Nenova Z., Nenov T., Nedev N., Machkova M. (2015). Humidity sensing elements based on cerium doped titania-silica thin films prepared via a sol–gel method. Sens. Actuators B Chem..

[14] Solanki M., Parihar U., Patel K., Jain V., Sharma S.S., Ray J. (2025). Titanium dioxide (TiO_2_) as a potential material in memristor for gamma (γ) ray detection. Chem. Phys. Impact.

[15] Yu S., Zhang H., Chen C., Lin C. (2019). Investigation of humidity sensor based on Au modified ZnO nanosheets via hydrothermal method and first principle. Sens. Actuators B Chem..

[16] Akçay N., Algün G., Öztel H.O. (2023). Fabrication of ultra-sensitive humidity sensors based on Ce-doped ZnO nanostructure with superfast response and recovery time. J. Mater. Sci..

[17] Modaresinezhad E., Darbari S. (2019). Realization of a room-temperature/self-powered humidity sensor, based on ZnO nanosheets. Sens. Actuators B Chem..

[18] Pan S., Gayathri G., Reshma T.S., Mangamma G., Prasad A.K., Das A. (2022). A sensitive humidity sensor at low pressure with SnO_2_ QDs. Sens. Actuators A Phys..

[19] Kumar A., Kumari P., Kumar M.S., Gupta G., Shivagan D.D., Bapna K. (2023). SnO_2_ nanostructured thin film as humidity sensor and its application in breath monitoring. Ceram. Int..

[20] Zhang H., Zhang H., Man J., Chen C. (2023). Preparation of high performance Fe-doped SnO_2_ humidity sensor and its application in respiration detection. Sens. Actuators A Phys..

[21] Lina W.-D., Hong R.-Y., Chuang M.-H., Wub R.-J., Chavali M. (2021). Enhanced performance of humidity sensor based on Gr/hollow sphere ZrO_2_ nanocomposites. Sens. Actuators A Phys..

[22] Wang J., Su M.Y., Qi J.-Q., Chang L.-Q. (2009). Sensitivity and complex impedance of nanometer zirconia thick humidity sensors. Sens. Actuators B Chem..

[23] Manikandan V. (2021). Real environment humidity-sensing ability of Nd-doped Fe_2_O_3_ sensor. Sens. Bio-Sens. Res..

[24] Khana M.U., Hassana G., Awais M., Bae J. (2020). All printed full range humidity sensor based on Fe_2_O_3_. Sens. Actuators A Phys..

[25] Neri G., Bonavita A., Galvagno S., Donato N., Caddemi A. (2005). Electrical characterization of Fe_2_O_3_ humidity sensors doped with Li^+^, Zn^2+^ and Au^3+^ ions. Sens. Actuators B Chem..

[26] Zhao H., Zhang T., Qi R., Dai J., Liu S., Fei T., Lu G. (2018). Humidity sensor based on solution processible microporous silica nanoparticles. Sens. Actuators B Chem..

[27] Nenova Z., Nenov T., Kozhukharov S., Nedev N. (2018). Humidity sensing elements based on Si-Bi-O surface layers prepared via a sol-gel method. IEEE Sens. J..

[28] Nenova Z., Kozhukharov S., Nenov T., Nedev N., Machkova M. (2016). Combined influence of titania and silica precursors on the properties of thin film humidity sensing elements prepared via a sol-gel method. Sens. Actuators B Chem..

[29] Sberveglieri G., Anchisini R., Murri R., Ercoli C., Pinto N. (1996). An Al_2_O_3_ sensor for low humidity content: Characterization by impedance spectroscopy. Sens. Actuators B Chem..

[30] Cheng B., Tian B., Xie C., Xiao Y., Lei S. (2011). Highly sensitive humidity sensor based on amorphous Al_2_O_3_ nanotubes. J. Mater. Chem..

[31] Cheng B., Ouyang Z., Tian B., Xiao Y., Lei S. (2013). Porous ZnAl_2_O_4_ spinel nanorods: High sensitivity humidity sensors. Ceram. Int..

[32] Wang W., Virkar A.V. (2004). A conductimetric humidity sensor based on proton conducting perovskite oxides. Sens. Actuators B Chem..

[33] Thakur S., Patil P. (2014). Rapid synthesis of cerium oxide nanoparticles with superior humidity-sensing performance. Sens. Actuators B Chem..

[34] Biswas P., Kundu S., Banerji P., Bhunia S. (2013). Super rapid response of humidity sensor based on MOCVD grown ZnO nanotips array. Sens. Actuators B Chem..

[35] Cardoso R., Sarapajevaite G., Korsun O., Cardoso S., Ilharco L. (2017). Microfabricated sol-gel relative humidity sensors for soil suction measurement during laboratory tests. Can. Geotech. J..

[36] Cardoso R., Sarapajevaite G., Korsun O., Cardoso S., Ilharco L. (2017). Sol-Gel Relative Humidity Sensors: Impact of electrode geometry on performance in soil suction measurements. J. Sens. Technol..

[37] Gong M.M., Li Y.S., Guo Y.N., Lv X., Dou X.C. (2018). 2D TiO_2_ nanosheets for ultrasensitive humidity sensing application benefited by abundant surface oxygen vacancy defects. Sens. Actuators B Chem..

[38] Li Z., Haidry A.A., Gao B., Wang T., Yao Z.J. (2017). The effect of Co-doping on the humidity sensing properties of ordered mesoporous TiO_2_. Appl. Surf. Sci..

[39] Azmer M.I., Aziz F., Ahmad Z., Raza E., Najeeb M.A., Fatima N., Bawazeer T.M., Alsoufi M.S., Shakoor R.A., Sulaiman K. (2017). Compositional engineering of VOPcPhO-TiO_2_ nano-composite to reduce the absolute threshold value of humidity sensors. Talanta.

[40] Cosentino I.C., Muccillo E.N.S., Muccillo R. (2003). Development of zirconia-titania porous ceramics for humidity sensors. Sens. Actuators B Chem..

[41] Poonia E., Mishra P.K., Kiran V., Sangwan J., Kumar R., Raib P.K., Tomer V.K. (2018). Aero-gel assisted synthesis of anatase TiO_2_ nanoparticles for humidity sensing application. Dalton Trans..

[42] Radja I., Tudorache F., Amrani B., Guezzoul M., Ech-Chergui A.N., Kadari A.S., Driss-Khodja K., Reddy M.R.V., Mehdi A., Bendeddouche C.K. (2025). Unveiling growth and characterization of alkali metals-doped SnS_2_ thin films for resistive humidity sensing. Sens. Actuators A Phys..

[43] Manjunatha K., Chethan B., Wu S.Y., Ubaidullah M., Al-Kahtani A.A., Dhakal T., Angadi V.J. (2024). Synthesis of Li doped MgFe_2_O_4_ nanoparticles for humidity sensor applications. Ceram. Int..

[44] Yin M., Yang F., Wang Z., Zhu M., Liu M., Xu X., Xu Z. (2017). A Fast Humidity Sensor Based on Li+ -Doped SnO_2_ One-Dimensional Porous Nanofibers. Materials.

[45] Na B., Guo C., Wang T., Zhang X., Huo L., Li L., Liu Y., Cheng X., Xu Y. (2023). Multifunctional and highly sensitive humidity sensor based on KCl/Sm_2_O_3_ nanoflowers with ultrafast response. Sens. Actuators B Chem..

[46] Xu T., Li R., Zhang T., Li Y., Zhang H., Yuan Z., Zhuo K. (2025). Fabrication of high-Performance humidity sensors based on KCl-modified GaN nanorods for respiratory monitoring. Microchem. J..

[47] Chen G., Si R., Wang C. (2021). Na_4_Ti_5_O_12_ based humidity sensor with excellent linear response over a wide humidity range. Mater. Lett..

[48] Srilarueang S., Sreejivungsa K., Thanamoon N., Jarernboon W., Thongbai P. (2024). Optimizing sintering conditions and microstructure for enhanced dielectric and humidity sensing properties of Na_1/3_Ca_1/3_Tb_1/3_Cu_3_Ti_4_O_12_ ceramics. Mater. Chem. Phys..

[49] Xuan X., Li L., Li T., Wang J., Yu Y., Wang C. (2022). Boosting the Humidity Performances of Na_0.5_Bi_x_TiO_3_ by Tuning Bi Content. Nanomaterials.

[50] Wang D., Lou Y., Wang R., Wang P., Zheng X., Zhang Y., Jiang N. (2015). Humidity sensor based on Ga_2_O_3_ nanorods doped with Na^+^ and K^+^ from GaN powder. Ceram. Int..

[51] Li T., Si R., Wang J., Wang S., Sun J., Wang C. (2019). Microstructure, colossal permittivity, and humidity sensitivity of (Na, Nb) co-doped rutileTiO_2_ ceramics. J. Am. Ceram. Soc..

[52] Dimitriev Y., Ivanova Y., Iordanova R. (2008). History of sol-gel science and technology (review). J. Univ. Chem. Technol. Met..

[53] Faustini M., Nicole L., Ruiz-Hitzky E., Sanchez C. (2018). History of Organic–Inorganic Hybrid Materials: Prehistory, Art, Science, and Advanced Applications. Adv. Func. Mater..

[54] Haas K.-H., Amberg-Schwab S., Rose K., Schottner G. (1999). Functionalized coatings based on inorganic–organic polymers (ORMOCER^®^s) and their combination with vapor deposited inorganic thin films. Surf. Coat. Technol..

[55] Haas K.-H., Wolter H. (1999). Synthesis, properties and applications of inorganic–organic copolymers (ORMOCER^®^s). Curr. Opin. Solid State Mater. Sci..

[56] García-Martínez J.-M., Collar E.P. (2024). Current and Future Insights in Organic–Inorganic Hybrid Materials. Polymers.

[57] Adam H., Lakshmipriya T., Gopinath S.C.B., Adam T., Salim E.T., Fakhri M.A., Ashok-Kumar T. (2024). Introduction to Hybrid Materials and Nanostructures. Hybrid-Nanomaterials.

[58] Sanchez C., Julián B., Belleville P., Popall M. (2005). Applications of hybrid organic–inorganic nanocomposites. J. Mater. Chem..

[59] Arya M., Heera S., Meenu P., Deepa K.G. (2024). Organic-inorganic hybrid materials and architectures in optoelectronic devices: Recent advancements. ChemPhysMater.

[60] Zhou K., Qi B., Liu Z., Wang X., Sun Y., Zhang L. (2024). Advanced Organic–Inorganic Hybrid Materials for Optoelectronic Applications. Adv. Func. Mater..

[61] Tomala R., Lukowiak A., Borak B., Chiappini A., Blanc W., Ferrari M., Bouajaj A., Ristic D., Taccheo S., Strek W. Glass photonic structures fabricated by sol-gel route. Proceedings of the SPIE 10683, Fiber Lasers and Glass Photonics: Materials Through Applications.

[62] Livage J., Ganguli D. (2001). Sol–gel electrochromic coatings and devices: A review. Sol. Energy Mater. Sol. Cells.

[63] Dahmouche K., Atik M., Mello N.C., Bonagamba T.J., Panepucci H., Aegerter M.A., Judeinstein P. (1997). Investigation of new ion-conducting ORMOLYTES: Structure and properties. J. Sol. Gel. Sci. Technol..

[64] Nunes P.J., Zając W., Styszko K., Pereira S., Fortunato E., de Zea Bermudez V., Fernandes M. (2025). Novel ormolytes for smart electrochromic windows for energy-efficient buildings. Solid State Ion..

[65] Judeinstein P., Sanchez C. (1996). Hybrid organic–inorganic materials: A land of multidisciplinarity. J. Mater. Chem..

[66] Copéret C., Héroguel F. (2017). Recent Advances in Surface Organometallic Chemistry. Applied Homogeneous Catalysis with Organometallic Compounds: A Comprehensive Handbook in Four Volumes.

[67] Coperet C., Lefebvre F., Basset J.-M. (2020). Surface organometallic chemistry. Catalysis from A to Z: A Concise Encyclopedia.

[68] Nivetha P., Siranjeevi R., Susmitha R., Sameera Shabnum S., Krishna Raj C., Benazir K., Saravanan A., Vickram A.S. (2025). A Comprehensive Review on Advances in Synthesis and Characterization of Nanocomposite: Current Status and Emerging Applications. Environ. Qual. Manag..

[69] Pierre A.C. (2020). Hybrid Organic–Inorganic and Composite Materials. Introduction to Sol-Gel Processing.

[70] Cerveau G., Corriu R.J.P., Framery E. (2001). Nanostructured Organic-Inorganic Hybrid Materials: Kinetic Control of the Texture. Chem. Mater..

[71] Innocenzi P., Brusatin G., Guglielmi M., Babonneau F. (2017). Structural Characterization of Hybrid Organic–Inorganic Materials. Handbook of Sol-Gel Science and Technology.

[72] Neetu T., Dinesh K. (2018). Fabrication of Porous Nanoceramic Materials Based on Sol-Gel Chemistry. Smart Ceramics: Preparation, Properties, and Applications.

[73] Salinas D., Pecchi G., Fierro J.L.G. (2016). K_2_O supported on sol-gel CeO_2_-Al_2_O_3_ and La_2_O_3_-Al_2_O_3_ catalysts for the transesterification reaction of canola oil. J. Molec. Catal..

[74] Atanasova P., Kozhukharov S., Milanes M. (2015). Evaluation of the buffering effect possessed by diluted diammonium hexanitrocerate solutions. Ann. Proceeds Univ. Rousse.

[75] Kozhukharov S., Nenova Z., Nenov T., Machkova M., Kozhukharov V. (2013). Influence of Ce(III)/Ce(IV)-supplements on the Characteristics of Humidity Sensors with TiO_2_ Films Prepared via a Sol-gel Method. Bol. Soc. Esp. Cerám. Vidrio.

[76] Sridharan V., Menendez J.C. (2010). Cerium (IV) ammonium nitrate as a catalyst in organic synthesis. Chem. Rev..

[77] Nair V., Balagopal L., Rajan R., Mathew J. (2004). Recent Advances in Synthetic Transformations Mediated by Cerium (IV) Ammonium Nitrate. Acc. Chem. Res..

[78] Nair V., Deepthi A. (2007). Cerium (IV) Ammonium Nitrate. A Versatile Single-Electron Oxidant. Chem. Rev..

[79] Musil M., Skopal F., Hajek M., Vavra A. (2018). Butanolysis: Comparison of potassium hydroxide and potassium tert-butoxide as catalyst for biodiesel preparing from rapeseed oil. J. Environ. Manag..

[80] Patel C.K., Banerjee S., Kant K., Sengupta R., Aljaar N., Malakar C.C. (2023). Roles of Alkali Metals tert-Butoxide as Catalysts and Activators in Organic Transformations. Asian J. Org. Chem..

[81] Rupp J.L.M., Solenthaler C., Gasser P., Muecke U.P., Gauckler L.J. (2007). Crystallization of amorphous ceria solid solutions. Acta Mater..

[82] Craciun R. (1998). Characterization of mixed amorphous/crystalline cerium oxide supported on SiO_2_. Solid State Ion..

[83] Sato T., Misawa M., Maruyama K., Itoh K. (2007). Preparation of TiO_2_–Na_2_O glass by sol–gel method and structural characterization. J. Non-Cryst. Sol..

[84] Eddy D.R., Permana M.D., Sakti L.K., Sheha G.A.N., Solihudin, Hidayat S., Takei T., Kumada N., Rahayu I. (2023). Heterophase Polymorph of TiO_2_ (Anatase, Rutile, Brookite, TiO_2_ (B)) for Efficient Photocatalyst: Fabrication and Activity. Nanomaterials.

[85] Scherrer P. (1918). Bestimmung der Grösse und der inneren Struktur von Kolloidteilchen mittels Röntgenstrahlen. Nachrichten Von Ges. Wiss. Göttingen Math.-Phys. Klasse.

[86] Fubini B., Bolis V., Bailes M., Stone F.S. (1989). The reactivity of oxides with water vapor. Solid State Ion..

[87] Traversa E. (1995). Ceramic sensors for humidity detection: The state-of the-art and future developments. Sens. Actuators B Chem..

[88] Nenov T., Yordanov S. (1996). Ceramic Sensors: Technology and Applications.

[89] Anderson J.H., Parks G.A. (1968). Electrical conductivity of silica gel in the presence of adsorbed water. J. Phys. Chem..

[90] Dickey E.C., Varghese O.K., Ong K.G., Gong D., Paulose M., Grimes C.A. (2002). Room temperature ammonia and humidity sensing using highly ordered nanoporous alumina films. Sensors.

[91] Shimizu Y., Arai H., Seiyama T. (1985). Theoretical studies on the impedance-humidity characteristics of ceramic humidity sensors. Sens. Actuators.

[92] Kennour S., Lamrani N., Chaouchi A., Lorgouilloux Y., Rguiti M., Courtois C. (2023). Humidity Sensing Properties of (Na_0.5_Bi_0.5_)_0.94_Ba_0.06_TiO_3_ Lead-Free Ferroelectrics Ceramics. Sci. Sinter..

[93] Si R., Xie X., Li T., Zheng J., Cheng C., Huang S., Wang C. (2020). TiO_2_/(K,Na)NbO_3_ Nanocomposite for Boosting Humidity-Sensing Performances. ACS Sens..

[94] Li L., Xuan X., Chen G., Ma Y., Chen C., Wang C. (2021). Hydrothermal preparation of Na_0.5_Bi_0.5_TiO_3_ nanospheres towards high humidity sensing response. Sens. Actuators B Chem..

[95] Mi Y., Li P. (2023). Preparation and performance of NaTaO_3_/TiO_2_ humidity sensors with high responsivity. Results Phys..

[96] Zhang Y., Zheng X., Zhang T. (2011). Characterization and humidity sensing properties of Bi_0.5_Na_0.5_TiO_3_–Bi_0.5_K_0.5_TiO_3_ powder synthesized by metal-organic decomposition. Sens. Actuators B Chem..

